# Impact of the Different Corneal Storage Flasks on Endothelial Cell Loss During Cultivation—A Retrospective Analysis

**DOI:** 10.3390/jcm14207165

**Published:** 2025-10-11

**Authors:** Tarek Safi, Carolin Marion Kolb-Wetterau, Stephanie D. Grabitz, Francesco Buonfiglio, Melissa Apel, Joanna Wasielica-Poslednik

**Affiliations:** 1 Department of Ophthalmology, University Medical Center of the Johannes Gutenberg University Mainz, 55131 Mainz, Germany; carolin.kolb-wetterau@unimedizin-mainz.de (C.M.K.-W.); stephanie.grabitz@unimedizin-mainz.de (S.D.G.); fbuonfig@uni-mainz.de (F.B.); melissa.apel@unimedizin-mainz.de (M.A.); joanna.wasielica-poslednik@unimedizin-mainz.de (J.W.-P.); 2 Eye Bank of Rhineland-Palatinate in Mainz, 55131 Mainz, Germany

**Keywords:** corneal transplantation, endothelial cell density (ECD), endothelial cell loss, organ culture, eye banking, cornea storage container, corneal holder

## Abstract

**Objectives:** To evaluate the impact of different corneal storage containers—with and without corneal holders—on endothelial cell density (ECD) and endothelial cell loss (ECL) during organ culture, following a temporary shortage of Böhnke Donor Corneal Holders at a German eye bank. **Methods:** A retrospective analysis was conducted on 383 human donor corneas cultured in six types of containers between January and September 2024 at the Eye Bank of Rhineland-Palatinate. ECD was measured at 6.0 ± 1.2 days (ECD1) and again at 14.9 ± 4.4 days (ECD2) after retrieval using standardized organ culture protocols with an inverted light microscope. Group 1 (G1) used the standard 50 mL Corning^®^ flask with the Böhnke corneal holder. Groups 2–6 used alternative containers, with or without corneal holders. ECL was defined as the difference between ECD2 and ECD1. **Results:** Mean overall ECD1 was 2478.3 ± 843.4 cells/mm^2^. G1 showed ECD1 < 2000 cells/mm^2^ in 29% of corneas and low ECL (−1%). The 60 mL Ratiolab^®^ flask with Cornea-Claw^®^ was the best alternative, showing the lowest incidence of ECD1 < 2000 cells/mm^2^ (7%) and no ECL. Containers without corneal holders, particularly the 100 mL Sterilin™ and 15 mL Cryogenic Tube^®^, had significantly higher rates of ECD1 < 2000 (40% and 75%) and greater ECL (9% and 14%). **Conclusions:** Container design, especially volume and the presence of corneal holders, significantly influences endothelial preservation. Especially a lack of corneal holders led to unacceptable endothelial cell loss. Eye banks should prioritize appropriate flask configurations to minimize tissue deterioration, particularly during supply shortages.

## 1. Introduction

Corneal disorders are among the leading causes of irreversible vision loss globally [1,2]. Due to the immune-privileged nature of the ocular environment, corneal transplantation demonstrates a relatively high rate of graft survival compared to other forms of solid tissue transplantation [3,4], becoming the most frequently performed tissue transplant procedure worldwide [5,6]. In Germany alone, more than 9000 procedures are performed annually, yet a significant shortage of donor corneas persists, with over 4500 patients remaining on the national waiting list by the end of 2020 [7]. This ongoing demand highlights the need to optimize every step of the transplantation process, from donor selection and tissue retrieval to surgical implantation and postoperative management. Despite decades of clinical experience and the presence of several guidelines regulating the transplantation process, no universally and internationally accepted standard exists for the overall procedure [8].

A critical determinant of graft survival is the functional integrity and density of endothelial cells, as over 95% of corneal transplants are performed as either penetrating keratoplasty or Descemet membrane endothelial keratoplasty (DMEK) [7]. Endothelial cell density (ECD) of grafts is influenced by various donor-related and non-donor-related factors, such as the timing of postmortem tissue retrieval [9,10].

To ensure graft success, proper preservation of donor corneas is essential. During this process, corneas are stored in specially designed flasks that maintain sterile conditions. Two different methods are used: hypothermic storage (short-term culture), in which the temperature control is set between 1 and 10 °C, and organ culture with a temperature between 30 and 37 °C. For each type of storage condition, the maximum duration is specified. For hypothermic storage, the maximum duration is 14 days, and for organ culture it is 34 days, including storage in the dehydration (dextran-free) and transport (dextran-containing) medium. Due to the extended storage time, organ culture has become the standard method in many European countries [11].

One critical, but less studied, factor is the cornea’s positioning within the culture flask. In 1991, Böhnke introduced a spoon-shaped corneal holder to position the tissue centrally, promoting medium circulation and preventing debris accumulation on the endothelium [12]. Since then, various culture flasks and corneal holders have been developed, but no comparative studies have assessed their impact on endothelial cell viability to date. At the Eye Bank of Rhineland-Palatinate and at most German eye banks, corneas are routinely preserved in organ culture media at 30–37 °C using the Corning^®^ CellBIND^®^ Surface Cell Culture Flask (Corning Incorporated—Life Sciences, Durham, NC, USA) with the Böhnke Donor Corneal Holder^®^ (Bausch & Lomb Incorporated, Eppelheim, Germany). However, a national supply shortage of the Böhnke Donor Corneal Holder caused by a pause in its production due to legislative reasons in June 2024 necessitated testing alternative flasks, both with and without corneal holders.

This study aimed to evaluate whether these alternative containers led to increased endothelial cell loss (ECL) or higher discard rates during cultivation. To our knowledge, this is the first study to compare different container types, including those with and without corneal holders, providing critical insights into optimizing corneal storage conditions for transplantation.

## 2. Materials and Methods

We retrospectively analyzed ECD development in human donor corneas stored in six different types of culture flasks, including the standard 50 mL cell culture flask with Böhnke corneal holder, as well as five alternative storage systems used due to a national shortage of the standard Böhnke corneal holders. The study included retrospectively corneas retrieved between January and September 2024 at the Eye Bank of Rhineland-Palatinate, Germany. Corneas with positive serological findings (HIV, HBV, HCV, or syphilis) were excluded from the analysis to ensure safety and standardization. Ethical review and approval were waived for this study, as it is a retrospective analysis in which only fully anonymized data obtained during routine diagnostics and therapy were analyzed. Informed consent was obtained from all subjects involved in the study.

ECD was assessed at two time points using an inverted light microscope (Leica Microsystems, Wetzlar, Germany). The measurements are carried out by experienced employees of the eye bank and consist of at least three images taken at three different locations, both centrally and paracentrally on the cornea. In addition to endothelial cell counts, necrotic areas were also assessed as part of the ECD evaluation, providing further information on endothelial integrity. The first endothelial cell density measurement (ECD1) was taken 6.0 ± 1.2 days after corneal retrieval, during storage in an incubator (37 °C; 4% CO_2_) in culture medium I without Dextran (PAN-Biotech GmbH, Aidenbach, Germany). Corneas meeting the quality threshold (ECD1 > 2000 cells/mm^2^) were selected for possible transplantation and longitudinal evaluation. The second endothelial cell density measurement (ECD2) was performed 14.9 ± 4.4 days after corneal retrieval, following a one-day transition in culture medium II containing 5% Dextran (PAN-Biotech GmbH, Aidenbach, Germany), in accordance with standard corneal preservation protocols for organ culture.

**Figure 1:**
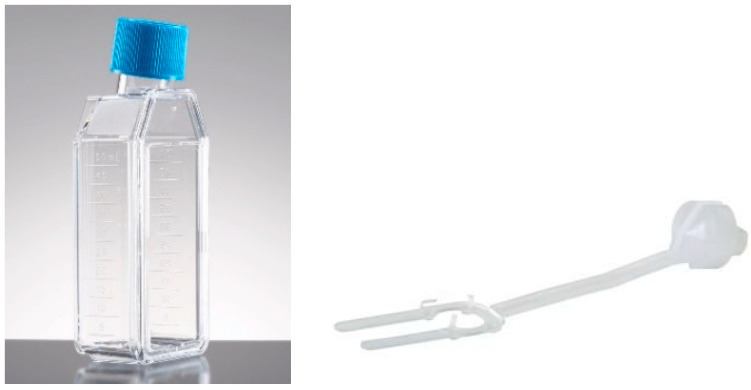
Group 1 (G1): 50 mL Corning^®^ CellBIND^®^ Surface Cell Culture Flask (Corning Incorporated—Life Sciences, Durham, NC, USA) with Böhnke donor corneal holder^®^ (Bausch & Lomb Incorporated, Eppelheim, Germany).

**Figure 2:**
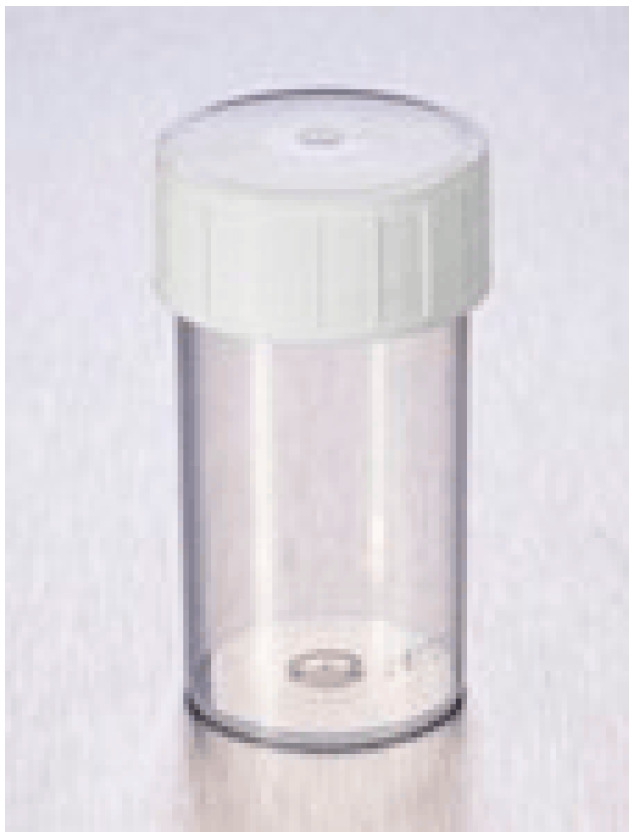
Group 2 (G2): 100 mL Sterilin™ Triple Bagged Polystyrene Containers^®^ (Thermo Fisher Scientific, Newport, UK) without corneal holder.

**Figure 3:**
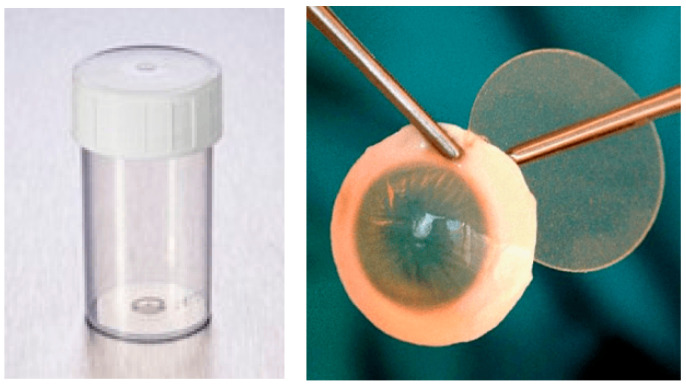
Group 3 (G3): 100 mL Sterilin™ Triple Bagged Polystyrene Containers^®^ (Thermo Fisher Scientific, Newport, UK) with Cornea-Claw^®^ (Hippocratech bv, Rotterdam, The Netherlands).

**Figure 4:**
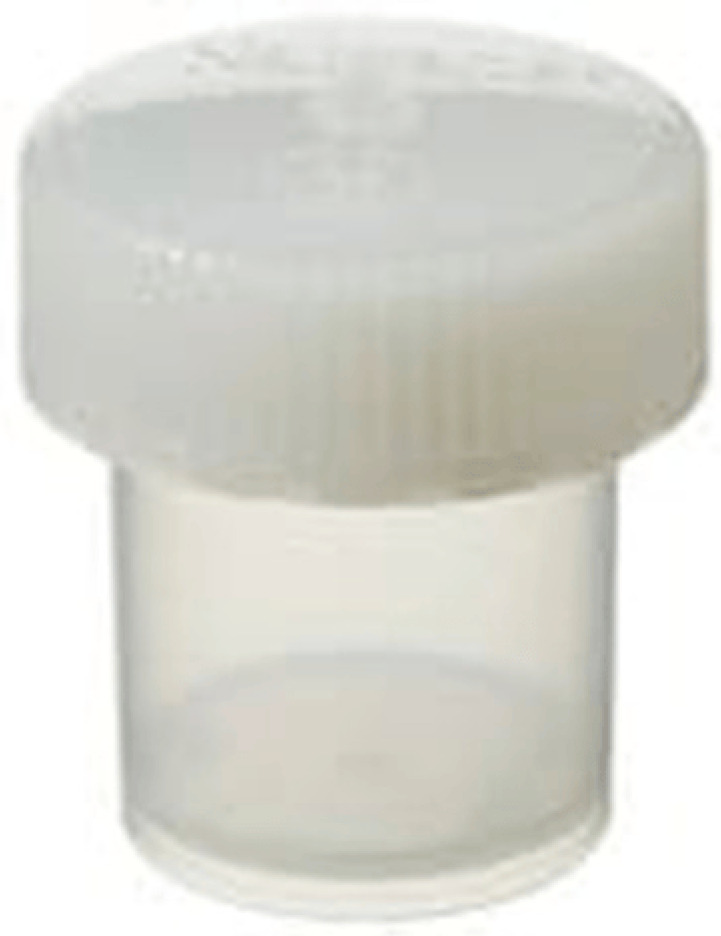
Group 4 (G4): 15 mL Nalgene™ General Long-Term Storage Cryogenic Tubes^®^ (Fisher Scientific GmbH, Schwerte, Germany) without corneal holder.

**Figure 5:**
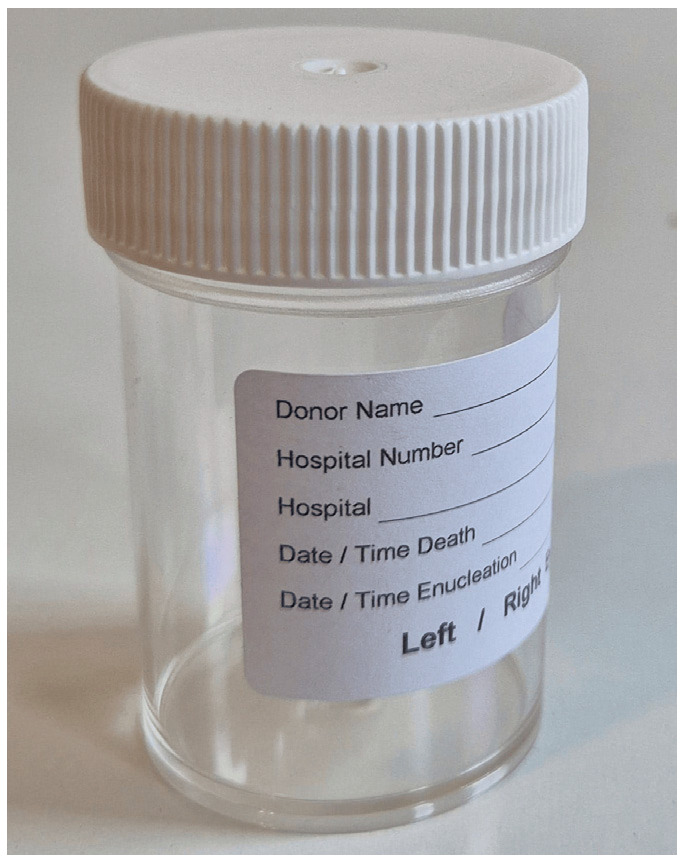
Group 5 (G5): 60 mL Eyeball Storage Pot^®^ (Medfor, Aldershot, Hampshire, GU12 4YD, UK) without corneal holder.

**Figure 6:**
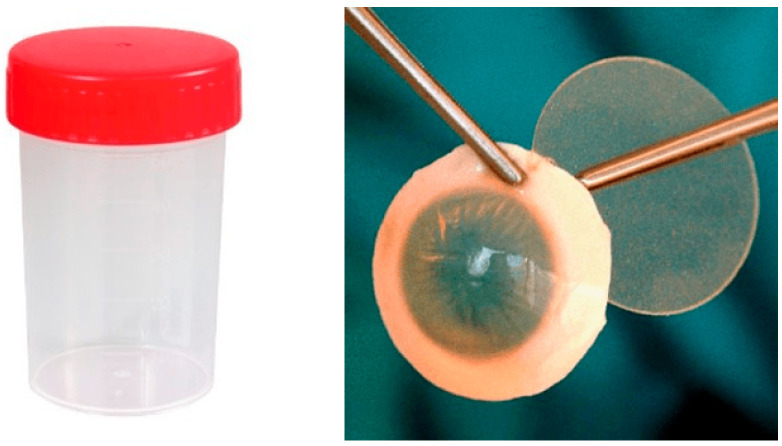
Group 6 (G6): 60 mL Multipurpose beakers^®^ (Ratiolab GmbH, Dreieich, Germany) with Cornea-Claw^®^ (Hippocratech bv, Rotterdam, The Netherlands).

The corneas were stored in the following types of containers, each forming a comparison group:

1. Standard container (Figure 1):

2. Alternative containers and holding systems (Figure 2, Figure 3, Figure 4, Figure 5 and Figure 6):

In addition to ECD values, the following variables were collected or derived: donor’s age, sex, lens status (phakic, pseudophakic, or aphakic), death-to-explantation interval (DEI) [hours], and ECL defined as the difference between ECD2 and ECD1 [cells/mm^2^ and percentages].

## 3. Statistical Analysis

Statistical analyses were performed using Microsoft Excel (Microsoft Corporation, Redmond, WA, USA). Descriptive data were expressed as mean ± standard deviation. The Chi-square test was used for comparison of categorical variables across groups, and the paired *t*-test was used to assess differences between ECD1 and ECD2 within each group. All statistical tests were two-tailed, with a significance level (α) set at 0.01 after Bonferroni correction.

## 4. Results

### 4.1. Demographics

A total of 383 corneas were included in this study. The mean donor age was 72.8 ± 11.6 years, with 61% male and 39% female donors. The mean DEI was 34.3 ± 17.2 h. Among the analyzed eyes, 212 were phakic, 169 were pseudophakic, and 2 were aphakic (1 in G1 and 1 in G3). A detailed summary of these baseline characteristics is provided in Table 1.

### 4.2. ECD1

At the time of ECD1 measurements performed 6.0 ± 1.2 days post-retrieval, 115 of 383 corneas (30%) demonstrated endothelial cell counts below the 2000 cells/mm^2^ threshold. These corneas were stratified across the six experimental groups as detailed in Table 2.

Comparative analysis against the standard flask with Böhnke holder (G1) revealed:

- G4 (15 mL without corneal holder) exhibited a significantly higher proportion of subthreshold corneas (*p* < 0.001).
- G6 showed a trend toward significance with a clinically relevant difference of 22% (*p* = 0.088).

### 4.3. ECD2

Corneas meeting the quality threshold (ECD1 > 2000 cells/mm^2^) were selected for longitudinal evaluation and for possible transplantation. These underwent a second ECD2 measurement performed 8.9 ± 4.1 days after the ECD1 assessment, and following a one-day transition in culture medium II containing 5% Dextran, the comparative analysis revealed:

- G1 and G6 demonstrated essentially stable endothelial counts.
- All other groups showed mild ECL without statistical significance (range: 160–327 cells/mm^2^).

However, these intergroup differences did not reach statistical significance (*p* > 0.01 for all comparisons). Complete longitudinal data are presented in Table 3.

**Figure 7:**
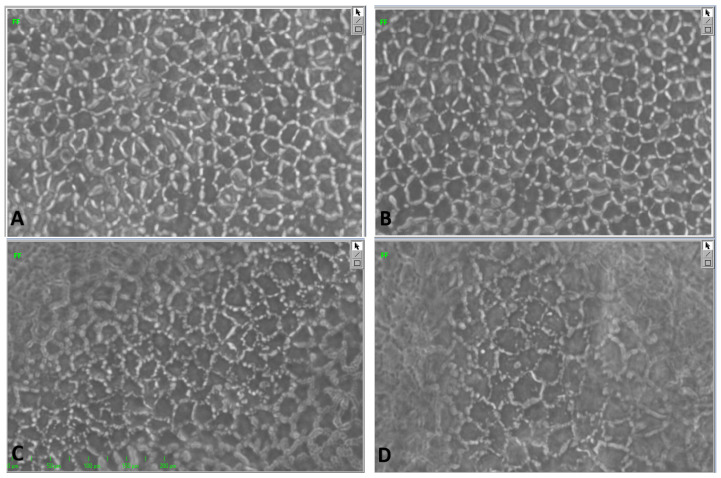
Endothelial cell images: (**A**) from G1 having an ECD1 of 2412 cells/mm^2^, (**B**) the same cornea having an ECD2 of 2329 cells/mm^2^, (**C**) from G4 having an ECD1 of 2124 cells/mm^2^, and (**D**) the same cornea having an ECD2 of 993 cells/mm^2^. (ECD1 = endothelial cell density at the first time point; ECD2 = endothelial cell density at the second time point, G1 = Group 1, G4 = Group 4).

Figure 7 shows a few examples of the endothelial cell images from G1 and G4 during ECD1 and ECD2 measurements.

We then assessed how many corneas with initial ECD1 values above 2000 cells/mm^2^ dropped below the transplant threshold of 2000 cells/mm^2^ at ECD2 (Table 4). Comparative analysis with the reference group (G1) revealed:

- Significantly higher rates of ECL to less than 2000 cells/mm^2^ in G4 (*p* = 0.008) and G5 (*p* = 0.001).
- No significant trend in G2 and G3 as well as no ECL in G6.

Table 5 represents a clear summary of both ECD and ECL measurements in each group.

## 5. Discussion

To our knowledge, our study represents the first evaluation of different corneal storage containers with and without corneal holders and their impact on endothelial cell density during organ culture. Our study was driven by a supply shortage of the standard flasks with Böhnke holders, compelling us to assess alternative systems. The findings provide evidence that container design significantly influences endothelial preservation, with important implications for graft quality and eye bank practices.

The standard 50 mL flask with Böhnke corneal holder (G1) demonstrated an expected performance, showing a rate of subthreshold ECD1 values (<2000 cells/mm^2^) of 29% and minimal endothelial cell loss during cultivation. In another study, we found 52% having an initial ECD >2000 cells/mm^2^ and 39% <2000 cells/mm^2^ (9% missing values) [10]. This reaffirms the well-established design advantages of the Böhnke corneal holder, possibly leading to enhanced nutrient circulation and reduced debris accumulation on the endothelium [12]. The study by Filev et al. reported an average ECD after retrieval of 2640 cells/mm^2^ with a loss of 17.5 cells/mm^2^ per day in organ culture, resulting in a slightly higher ECD than in our study measured at day 6 after retrieval [13]. However, in their study, the donor age was lower (66.1 years) than in our study, which may have an impact on initial ECD. According to Schön et al., corneas lose 6.2 cells/mm^2^ per year [10]. Among alternatives, the 60 mL Ratiolab flask with Cornea-Claw^®^ (G6) emerged as a particularly promising option, showing even better outcomes than G1 with only 7% donor corneas falling below the 2000 cells/mm^2^ threshold at ECD1. This suggests that certain modern holder systems may match or potentially exceed traditional solutions. The superior results of G6 (60 mL Ratiolab^®^ flask with Cornea-Claw^®^) may be attributed to the more favorable balance between container volume and stabilization. The intermediate size of the flask likely reduced turbulence compared to the 100 mL systems, while the Cornea-Claw^®^ ensured central positioning and prevented direct endothelial contact with the container bottom. This combination appears to have provided optimal conditions for nutrient exposure and mechanical protection, which may explain why G6 achieved the best endothelial preservation in our series. However, compared with G1, corneas preserved in G6 had a shorter DEI of 23.2 ± 14.2 h vs. 35.8 ± 16.6 h, a lower mean age of 68 ± 12 years vs. 73 ± 11 years, and a greater proportion of phakic eyes of 10/4 (2.5) vs. 165/117 (1.4), making larger studies essential to confirm the benefits of this flask.

In contrast, a system with low-medium volume and a lack of corneal stabilization, like the 15 mL Cryogenic Tube^®^ without a holder (G4), performed poorly. G4 showed alarmingly high rates of inadequate ECD1, with 75% of corneas presenting ECD1 lower than 2000 cells/mm^2^ already after 6 days of culture, and significant decline towards the end of the culture phase (ECD2-ECD1 of −50%; *p* = 0.008). However, it should be noted that there were only 4 corneas in this group. Although the 60 mL Medfor container without holder (G5) demonstrated satisfying ECD1, with only 19% having an ECD1 < 2000 cells/mm^2^, a significant amount of 38% drops to subthreshold levels at ECD2 (*p* = 0.001), which may be partially caused by lower ECD1 compared to the other groups. These outcomes likely reflect compromised tissue positioning on the bottom of the flask, leading to uneven nutrient exposure and increased mechanical stress. This effect appears to be more pronounced when the medium volume is critically low.

The 100 mL Thermo Fisher flasks (G2 without holder/G3 with Cornea-Claw^®^) showed intermediate results, with the Cornea-Claw^®^ version (G3) showing a trend toward a significantly high ECL (*p* = 0.097). The comparable outcomes observed between G2 and G3 may be explained by the relatively large volume of the 100 mL Sterilin™ flask, which likely resulted in increased medium turbulence and reduced stability of the corneal position, thereby diminishing the potential advantage of the Cornea-Claw^®^. Furthermore, G3 included a higher proportion of pseudophakic donors compared to G2, a factor that is known to negatively affect endothelial cell density and may have further contributed to the lack of difference between these groups.

The study highlights a critical gap in current guidelines. While the “Guide to the Quality and Safety of Tissues and Cells” and “Technical Guidelines for Ocular Tissue” detail retrieval and cultivation protocols, they lack specific recommendations about container types or holder use [14,15]. Our data demonstrate that both stabilization methods and flask dimensions significantly impact endothelial preservation. Larger containers (e.g., 100 mL) showed a trend to higher discard rates, possibly due to increased fluid turbulence, while corneas in small flasks (15 mL) without any holder performed the poorest.

Several limitations warrant consideration. The retrospective design and unequal group sizes (with G1 predominating) may affect generalizability. This limitation resulted from the retrospective design of our study and the limited availability of corneas stored in alternative flasks during the temporary shortage and led to having small sample sizes in the comparison groups. From a methodological perspective, a larger control group increases the precision of baseline estimates and enhances the robustness of comparisons, even when experimental groups are smaller. While we controlled for storage medium consistency and excluded serologically unsuitable tissue, unmeasured confounders like donor endothelial health or handling variations could persist. There was a much higher rate of pseudophakic donors in G3, G4, and G5, negatively influencing the ECD [16]. This was also demonstrated by Schön et al., who showed that corneas from pseudophakic eyes had on average 375 cells/mm^2^ less than those from phakic eyes (confidence interval −460.4 to −294.5, *p* < 0.001) [10]. Furthermore, ECD1 was performed in culture medium M1, whereas ECD2 was performed in culture medium M2, which may account for differences between the two measurements due to varying medium compositions and their potential effects on cell morphology and visibility [17,18]. Furthermore, our study focused on organ culture and did not evaluate the performance of containers for hypothermic storage. Future prospective studies should incorporate more morphological and optical parameters to achieve a more comprehensive assessment of preservation efficacy.

Future research should prospectively validate these findings and investigate optimal holder-flask interactions. The development of standardized container design guidelines could significantly improve preservation outcomes across eye banks. Our results confirm that endothelial preservation depends not only on biological factors and media composition but also on the mechanical stability and spatial configuration provided by the storage system.

## 6. Conclusions

This study confirms that corneal storage systems significantly affect endothelial preservation. When the standard 50 mL flasks with Böhnke holders were unavailable, alternatives showed marked performance differences. The 60 mL flask with Cornea-Claw^®^ proved most reliable among the alternatives, minimizing cell loss and maintaining transplant-ready tissue. Conversely, a small 15 mL container with the donor cornea lying on its bottom led to unacceptable endothelial deterioration. Proper tissue fixation and optimal flask volume are thus critical for viability. While supply issues may require temporary alternatives, eye banks should prioritize systems ensuring stabilization and adequate medium volume. These findings highlight that container design merits equal consideration as other quality measures in corneal preservation. Standardizing storage systems could further enhance global donor cornea cultivation.

## Figures and Tables

**Table 1:** Descriptive and demographic results of donor corneas cultured in different types of containers (G1–G6).

Groups	Total Cornea Count	Death-to-Explantation Interval [hour]	Mean Age [year]	Male/ Female	Phakic/ Pseudophakic
G1	283	35.8 ± 16.6	73 ± 11	176/107	165/117
G2	10	29.5 ± 16.3	72 ± 10	8/2	6/4
G3	44	30.0 ± 19.5	72 ± 13	27/17	23/20
G4	16	39.2 ± 21.0	79 ± 5	4/12	4/12
G5	16	26.2 ± 10.5	75 ± 9	6/10	4/12
G6	14	23.2 ± 14.2	68 ± 12	8/6	10/4
Total	383	34.3 ± 17.3	73 ± 11	229/154	212/169

**Table 2:** Analysis of endothelial cell density at the first time point (ECD1) in different types of alternative containers (G2-G6) compared to standard G1.

Groups	Total Cornea Count	Corneal Count with ECD1 > 2000 cells/mm^2^	Corneal Count with ECD1 < 2000 cells/mm^2^ (%)	Chi-Square Test Compared with G1
G1	283	201	82 (29%)	-
G2	10	6	4 (40%)	0.451
G3	44	31	13 (30%)	0.938
G4	16	4	12 (75%)	<0.001
G5	16	13	3 (19%)	0.377
G6	14	13	1 (7%)	0.075

**Table 3:** Endothelial cell loss (ECL) is defined as the difference between endothelial cell density at the first time point (ECD1) and endothelial cell density at the second time point (ECD2) in different containers (G1-G6).

Groups	Total Cornea Count	Mean ± SD ECD1 [cells/mm^2^]	Mean ± SD ECD2 [cells/mm^2^]	Mean ± SD ECL [cells/mm^2^]	ECL [%]	*t*-Test
G1	283	2480 ± 637	2460 ± 544	−20 ± 552	−1%	0.125
G2	10	2441 ± 360	2217 ± 463	−223 ± 473	−9%	0.397
G3	44	2514 ± 657	2308 ± 839	−206 ± 795	−8%	0.127
G4	16	2420 ± 570	2093 ± 396	−327 ± 446	−14%	0.282
G5	16	2351 ± 514	2191 ± 554	−160 ± 509	−7%	0.418
G6	14	2569 ± 381	2613 ± 245	44 ± 254	2%	0.597

**Table 4:** Analysis of the corneas that dropped from ECD1 > 2000 cells/mm^2^ to ECD2 < 2000 cells/mm^2^ (ECD1 = endothelial cell density at the first time point; ECD2 = endothelial cell density at the second time point) in alternative containers (G2–G6) compared to standard G1.

Groups	Corneal Count with ECD1 > 2000 cells/mm^2^	Corneal Count with ECD2 < 2000 cells/mm^2^ (%)	Chi-Square Test Compared with G1
G1	201	19 (9%)	-
G2	6	1 (17%)	0.556
G3	31	6 (19%)	0.097
G4	4	2 (50%)	0.008
G5	13	5 (38%)	0.001
G6	13	0 (0%)	0.245

**Table 5:** Summary of ECD measurements and ECL in each group (ECD1 = endothelial cell density at the first time point; ECD2 = endothelial cell density at the second time point; ECL = endothelial cell loss).

Groups		Total Cornea Count	ECD1 < 2000 cells/mm^2^	ECL = ECD2 − ECD1 cells/mm^2^	ECD2 < 2000 cells/mm^2^
G1	50 mL Böhnke corneal holder	283	29%	−1%	9%
G2	100 mL no holder	10	**40%**	−9%	17%
G3 *	100 mL Cornea-Claw^®^	44	30%	−8%	19%
G4 *	**15 mL** **no holder**	16	**75%**	**−14%**	**50%**
G5 *	60 mL no holder	16	19%	−7%	**38%**
*G6*	*60 mL* *Cornea-Claw^®^*	14	*7%*	*+2%*	*0%*

* Groups with higher numbers of pseudophakic donors; bold—particularly pronounced endothelial cell loss; italic—particularly promising alternative to G1.

## Data Availability

The data presented in this study are available on request from the corresponding author.
